# The significant scale up and success of Transmission Assessment Surveys '*TAS*' for endgame surveillance of lymphatic filariasis in Bangladesh: One step closer to the elimination goal of 2020

**DOI:** 10.1371/journal.pntd.0005340

**Published:** 2017-01-31

**Authors:** A. K. M. Shamsuzzaman, Rouseli Haq, Mohammad J. Karim, Motasim B. Azad, A. S. M. Sultan Mahmood, Abul Khair, Muhammad Mujibur Rahman, Israt Hafiz, K. D. Ramaiah, Charles D. Mackenzie, Hayley E. Mableson, Louise A. Kelly-Hope

**Affiliations:** 1 Filariasis Elimination and STH Control Program, Ministry of Health and Family Welfare, Communicable Disease Control, Directorate General of Health Services, Dhaka, Bangladesh; 2 Consultant on Lymphatic Filariasis, Tagore Nagar, Pondicherry, India; 3 Department of Parasitology, Liverpool School of Tropical Medicine, Liverpool, United Kingdom; 4 Department of Pathobiology, Michigan State University, East Lansing, Michigan, United States of America; Washington University School of Medicine, UNITED STATES

## Abstract

**Background:**

Bangladesh had one of the highest burdens of lymphatic filariasis (LF) at the start of the Global Programme to Eliminate Lymphatic Filariasis (GPELF) with an estimated 70 million people at risk of infection across 34 districts. In total 19 districts required mass drug administration (MDA) to interrupt transmission, and 15 districts were considered low endemic. Since 2001, the National LF Programme has implemented MDA, reduced prevalence, and been able to scale up the WHO standard Transmission Assessment Survey (TAS) across all endemic districts as part of its endgame surveillance strategy. This paper presents TAS results, highlighting the momentous geographical reduction in risk of LF and its contribution to the global elimination target of 2020.

**Methodology/Principal findings:**

The TAS assessed primary school children for the presence of LF antigenaemia in each district (known as an evaluation unit—EU), using a defined critical cut-off threshold (or ‘pass’) that indicates interruption of transmission. Since 2011, a total of 59 TAS have been conducted in 26 EUs across the 19 endemic MDA districts (99,148 students tested from 1,801 schools), and 22 TAS in the 15 low endemic non-MDA districts (36,932 students tested from 663 schools). All endemic MDA districts passed TAS, except in Rangpur which required two further rounds of MDA. In total 112 students (male n = 59; female n = 53), predominately from the northern region of the country were found to be antigenaemia positive, indicating a recent or current infection. However, the distribution was geographically sparse, with only two small focal areas showing potential evidence of persistent transmission.

**Conclusions/Significance:**

This is the largest scale up of TAS surveillance activities reported in any of the 73 LF endemic countries in the world. Bangladesh is now considered to have very low or no risk of LF infection after 15 years of programmatic activities, and is on track to meet elimination targets. However, it will be essential that the LF Programme continues to develop and maintain a comprehensive surveillance strategy that is integrated into the health infrastructure and ongoing programmes to ensure cost-effectiveness and sustainability.

## Author summary

Lymphatic filariasis (LF) was highly endemic in Bangladesh at the start of the Global Programme to Eliminate Lymphatic Filariasis (GPELF) in 2000, with approximately 70 million people at risk. To address this burden, the National LF Programme implemented mass drug administration (MDA) in 19 highly endemic districts to interrupt transmission, and conducted sentinel site assessments in 15 low endemic districts. In 2011, as part of the LF Programme’s endgame surveillance strategy, the standard WHO Transmission Assessment Survey (TAS) was used to show the reduction below transmission thresholds in order to stop MDA in all 34 endemic districts by testing a total of 136,080 primary school-aged children in 2,464 schools using rapid diagnostic tests. The data show that LF transmission has been interrupted in all districts except one, with the latter requiring two further two rounds of MDA. Bangladesh can now be considered, after 15 years of LF programmatic activities, as having very low or no risk of LF infection and is therefore on track to meet National and Global elimination targets of 2020.

## Introduction

Bangladesh is a remarkable example in terms of the progress it has made in the elimination of lymphatic filariasis (LF), following the launch of the Global Programme to Eliminate LF (GPELF) by the World Health Organization (WHO) in 2000 [1]. Bangladesh was one of the first countries in the South-East Asia Region to start the elimination process with mass drug administration (MDA) to interrupt transmission in endemic areas [2,3], and one of the first countries to begin the elimination verification process using the new WHO guidelines of the Transmission Assessment Survey (TAS) on a large scale [4,5]. Bangladesh was considered to be widely endemic for LF, caused by the parasite *Wuchereria bancrofti* and transmitted by *Culex* sp. mosquitoes [2,3,6]. An estimated 70 million people (approximately half the total population) were considered to be at risk of LF infection, with tens of thousands of people suffering from various forms of clinical presentation, including limb lymphoedema/elephantiasis and hydrocele [7,8].

Fortunately, the Directorate General of Health Services in Bangladesh recognised the immense burden of LF, and responded positively to the new GPELF initiative [1]. In 2001, the Ministry of Health and Family Welfare launched the National LF Elimination Programme with an aim to eliminate the disease as a public health problem by 2020, with MDA and morbidity management as its core components [2,3]. The decision to classify districts as endemic and implement MDA was made in the early phase of the Global and National LF Elimination Programmes, and not always straightforward. Therefore, the Bangladesh LF Programme adopted a conservative approach and used a combination of the following three parameters to inform MDA eligibility; i) microfilaria (Mf) prevalence, ii) antigenaemia (Ag) prevalence, and iii) frequency of clinical cases.

**Fig 1:**
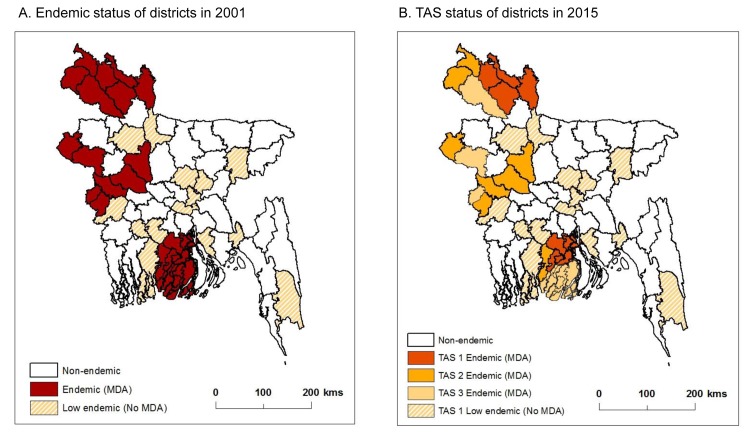
Endemicity and TAS status of districts. A. Endemic status of districts in 2001. B. TAS status of districts in 2015.

Baseline prevalence mapping and historical data indicated that the disease was endemic in 19 of the 64 districts and were considered eligible for MDA due to Ag and Mf rates of between 1% and 17% and evidence of clinical cases [9–11]. Some endemic districts, however, showed <1.0% Mf rate, but had some localised areas with chronic disease patients. Hence, it was decided to implement MDA, as a very conservative approach. Overall, three broad regions of the country required MDA, which included districts from the north (Rangpur Division) central west (Rajshahi/Khulna Divisions) and the south (Barisal Division) as shown in Fig 1A. The burden is highest in Rangpur Division, where 23% Mf prevalence and up to 10% chronic disease have been reported [3,12]. An additional 15 districts across the central and southern regions were considered to have low endemicity and not requiring MDA (Fig 1A), though some districts had considerable Ag positivity, their Mf rates were 0% and clinical cases were rare. Hence the programme decided not to implement MDA.

**Fig 2:**
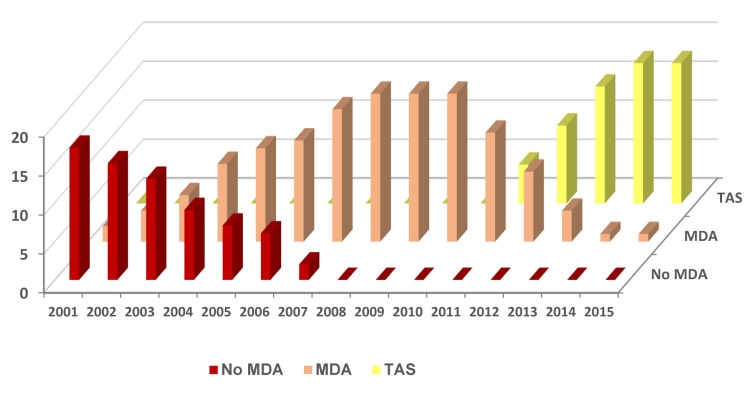
Endemic districts scaling up and down MDA and TAS activities.

In 2001, the Bangladesh LF Elimination Programme began MDA in a single district using the drugs albendazole (procured from GSK through WHO), and diethylcarbamazine (DEC; procured locally using the standard government drug policy) and has since steadily scaled up to reach full geographical coverage i.e. all 19 endemic districts (Fig 2), targeting approximately 35 million people with door-to-door distribution [3,13]. Each endemic district was considered to be an implementation unit (IU) for MDA with implementation conducted in November every year using health workers and community volunteers with a ratio of one volunteer to approximately 1000 people. By 2010 the LF Programme had successfully distributed at least three rounds of MDA to all 19 endemic districts, with 12 districts receiving more than six rounds of MDA accounting for more than 150 million treatments to the 35 million target population [13,14]. Overall reported treatment rates have been high, which were confirmed by independent coverage surveys, in accordance with the WHO guidelines i.e. at least five rounds of MDA and 65% coverage of the total population in an endemic area [15]. This is considered to be sufficient to interrupt transmission, and provides the basis to commence pre-TAS and TAS activities.

Ongoing assessments through sentinel site surveillance have been crucial to measuring the impact of MDA, and determining when to stop this aspect of the programme [2]. In 2010, the LF Programme identified 10 districts that could potentially stop MDA based on Mf rates of <1%, which coincided with the development of the new TAS guidelines [15]. Bangladesh was one of the first countries to consider the new WHO TAS protocol to verify the interruption of LF transmission following MDA [13]. The Bangladesh Health Services with the support from two major international donors were able to support steadily scale up TAS following MDA in the 19 endemic districts between 2010 and 2015 (Table 1,Fig 2). Further, the LF Programme has taken the initiative to assess the 15 low endemic districts (not requiring MDA) using the TAS method following the recommendation of the WHO South East Asian Regional Programme Review Group, with activities starting in 2014 [14,16]. The TAS protocol was used as there is currently no recommended strategy for assessing low endemic districts, which remains an important issue for the GPELF to address as many countries make good progress towards the endgame [17].

**Table 1:** Schedule for TAS (and Pre-TAS) Surveys.

No.	District	Pre-TAS (Mf survey)	TAS 1	TAS 2	TAS 3
1.	Meherpur	2011	2011	2013	2015
2.	Barguna	2011	2011	2013	2015
3.	Patuakhali	2011	2011	2013	2015
4.	Dinajpur–EU A	2011	2011	2013	2015
	Dinajpur–EU B	2011	2011	2013	2015
5.	Rajshahi–EU A	2011	2011	2013	2015
	Rajshahi–EU B	2011	2011	2013	2015
6.	Sirajganj–EU A	2012	2012	2015	2017
	Sirajganj–EU B	2012	2012	2015	2017
7.	Pabna–EU A	2012	2012	2015	2017
	Pabna–EU B	2012	2012	2015	2017
8.	Kushtia	2012	2012	2015	2017
9.	Chuadanga	2012	2012	2015	2017
10.	Pirojpur	2012	2012	2015	2017
11.	Chapainawabganj	2013	2013	2015	2017
12.	Panchagarh	2013	2013	2015	2017
13.	Thakurgaon	2013	2013	2015	2017
14.	Barisal–EU A	2013	2013	2016	2018
	Barisal–EU B	2013	2013	2016	2018
15.	Jhalokathi	2013	2013	2016	2018
16.	Rangpur–EU A	2014	2014*	2018	2020
		2016	2016		
	Rangpur–EU B	2014	2014*	2018	2020
		2016	2016		
17.	Kurigram–EU A	2014	2014	2016	2018
	Kurigram–EU B	2014	2014	2016	2018
18.	Lalmonirhat	2014	2014	2016	2018
19.	Nilphamari	2014	2014	2016	2018

* TAS fail–MDA x2 rounds.

Grey–remaining TAS activities until 2020.

Since the publication of the WHO TAS guidelines, a number of international workshops have been conducted [18], and more than one third of endemic countries have started to use them, with mixed results and implications [5,19,20]. Given that Bangladesh was one of the first countries to implement this TAS protocol and has been successful at expanding it on such a large scale, this paper presents the results of TAS across both the endemic and low endemic districts, highlighting the significant geographical reduction in risk, reasons for success and its contribution to the national, South-East Asia regional and global elimination targets of 2020 [2–4,21].

## Methods

### Endemic districts (required MDA)

**Fig 3:**
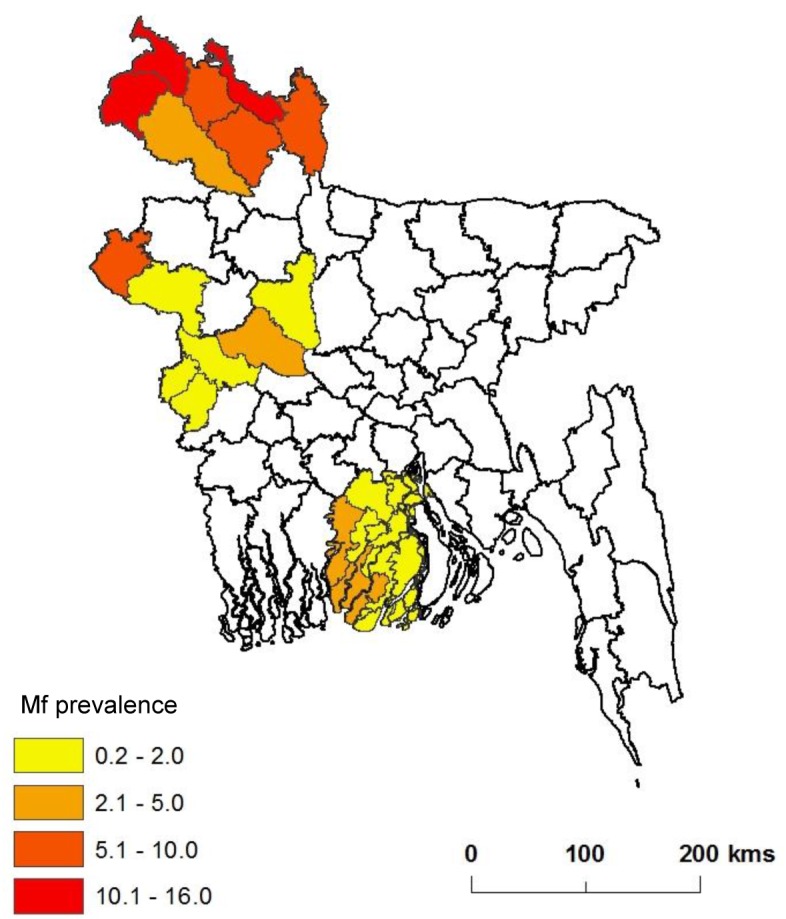
Baseline prevalence in endemic districts in 2001.

The endemic districts from the northern region include Dinajpur, Kurigram, Lalmonirhat, Nilphamari, Panchagarh, Rangpur and Thakurgaon with baseline Mf prevalence rates ranging from 4.8% to 16%, and number of MDA rounds from 5 to 12 (Table 2). The majority of these districts were the first to start MDA (before 2005) and last to start TAS (after 2011). This contrasts to the central western and southern regions where prevalence rates were lower and districts received fewer number of MDA rounds. The endemic districts from the central west include Chuadanga, Kushtia, Meherpur, Chapainawabganj, Pabna, Rajshahi and Sirajganj with baseline Mf prevalence rates ranging from 0.2% to 8.4%, and number of MDA rounds from 5 to 9. The endemic districts from the south include Barguna, Barisal, Jhalokathi, Patuakhali and Pirojpur with baseline Mf prevalence rates ranging from 1.0% to 2.2%, and five MDA rounds conducted for all districts. An annual timetable of the pre-TAS and TAS schedule is outlined in Table 1, and the TAS status in 2015 mapped in Fig 1B and baseline prevalence rates in Fig 3, highlighting the three different regions.

**Table 2:** Summary measures and TAS 1–3 results for the 19 endemic districts (received MDA).

Districts	Pop. in million 2011 census	Baseline Mf %	MDA start year	No. MDA round	Coverage Range**	Pre-TAS Mf result	Sample size for each EU*	No. of schools tested per EU*	No of ICT +ve TAS 1	No of ICT +ve TAS 2	No of ICT +ve TAS 3	Total No of ICT +ve
**TAS 1 start 2011**												
Dinajpur-A	2.97	4.8	2005	5	89.7–96.9	0	1692	31	1	5	1	7
Dinajpur-B			2005	5		0	1692	30	6	5	1	12
Meherpur	0.66	1.0	2004	6	87.6–93.8	0	1556	30	0	0	0	0
Rajshahi-A	2.57	1.0	2005	5	82.0–91.9	0	1692	30	1	0	0	1
Rajshahi-B			2005	5		0	1692	30	0	4	0	4
Patuakhali	1.52	2.0	2005	5	87.1–96.8	0	1692	31	0	0	0	0
Barguna	0.88	2.8	2005	5	94.5–95.4	0	1692	33	1	1	0	2
**TAS 1 start 2012**												
Sirajganj -A	3.07	1.2	2006	6	79.4–92.7	0	1692	30	1	0	-	1
Sirajganj -B			2006	6		0	1692	31	0	0	-	0
Pabna-A	2.50	2.4	2007	5	88.4–99.0	0	1692	31	0	0	-	0
Pabna-B			2007	5		0	1692	30	3	0	-	3
Kushtia	1.93	0.2	2007	5	86.0–96.3	0	1692	30	0	0	-	0
Chuadanga	1.12	0.8	2007	5	78.1–96.0	0	1692	30	0	0	-	0
Pirojpur	1.10	2.2	2007	5	57.8–93.4	0	1692	36	2	0	-	2
**TAS 1 start 2013**												
Chapainawabganj	1.63	8.4	2004	9	82.6–98.1	0	1692	30	4	0	-	4
Panchagar	0.98	10.8	2001	12	75.1–95.8	0	1692	30	0	0	-	0
Thakurgaon	1.38	16.0	2002	11	74.9–93.6	0	1692	30	3	0	-	3
Barisal–A	2.29	1.0	2008	5	79.9–92.9	0	1692	30	0	0	-	0
Barisal–B			2008	5		0	1692	30	0	0	-	0
Jhalokathi	0.59	1.0	2008	5	74.4–98.3	0	1556	30	0	1	-	1
**TAS 1 start 2014**												
Nilphamari	1.82	10.0	2002	12	80.5–99.7	0	1692	30	9	5	-	14
Kurigram–A	2.05	6.0	2004	10	78.8–92.3	0	1692	30	2	0	-	2
Kurigram—B			2004	10		0	1692	30	0	0	-	0
Lalmonirhat	1.25	16.0	2002	12	82.6–94.1	0	1692	30	8	4	-	12
Rangpur–Aƚ	2.86	10.0	2005	9	66.3–95.9	0.1	1692	30	11	3	-	14
Rangpur–Bƚ			2005	9		0.05	1692	30	27	3	-	30
**TOTAL**	**33.17**	**-**	**-**	**-**		**-**	99,148*	1801*	**79**	**31**	**2**	**112**

** District reported and verified coverage rates for years 2009–2014 in Supporting information S1 File.

* Number of students and school tested per EU for each TAS activity. The total includes TAS 1, 2 and 3.

ƚ Rangpur–A and B TAS 1 repeated in 2016 after two rounds of MDA in 2014 and 2015.

### Low endemic districts (not requiring MDA)

The 15 low endemic districts, which did not require MDA due to the overall low endemicity evident from historical data and found during Ag sentinel site mapping activities in 2002–2004 included Bagerhat, Bandarban, Bogra, Feni, Gazipur, Gopalganj, Habiganj, Jamalpur, Jhenaidaha, Laxmipur, Munshiganj, Narayanganj, Narail, and Narshingdi, and peri-urban areas of Dhaka. The TAS in these low endemic districts were conducted throughout 2014 and 2015. The distribution of these districts is shown in Fig 1B.

### Selecting schools and sampling

The TAS activities for both the 19 endemic districts and 15 low endemic districts were implemented according to the guidelines of the WHO [15]. Briefly, the survey design, number of schools and number of students to be sampled, and the critical cut-off point (i.e. the number of positive cases found that determined if an evaluation unit (EU) passed or failed the TAS) were determined using the Survey Sample Builder (SSB;http://www.ntdsupport.org/resources/transmission-assessment-survey-sample-builder), which is specifically designed for TAS.

The SSB identified a cluster survey design as the most appropriate method for Bangladesh given its very high enrolment (≥ 90%) of students in primary school. The target population included students from the 1^st^ and 2^nd^ grades, most of whom were 6 to 7 years of age. The SSB generated random numbers in order to select the survey schools, as well as the students within each of the selected survey schools. The inputs for the SSB included the total number of primary schools for each EU, the total number of students enrolled in the 1^st^ and 2^nd^ grades in EU and the expected non-response rate. The data on the list of schools and numbers of students were obtained from the Directorate of Primary Education, Dhaka.

All the schools within the district were listed and ordered geographically, and then allocated serial numbers. The schools bearing the numbers that matched the random numbers (generated by SSB) were selected for the TAS. The list of selected survey schools was prepared and forwarded to the Bangladesh Ministry of Health Services and Ministry of Education, along with a request to the latter to write to each of the selected schools asking them to consent and participate in the survey, and inform parents of the upcoming activity. The Health Services then proceeded to forward the list of the survey schools to each of the district and sub-district managers and medical officers of the local Public Health Centres to notify them of the upcoming TAS activity.

### Consent procedures and ethical approval

The TAS activities are part of routine LF programme activities conducted by the Ministry of Health and Family Welfare and permit the use of oral consent for all surveys. Approval from the Ministry of Education, local school authorities and Head Masters of the selected school was obtained prior to the surveys. Parents and children were verbally informed of the procedures and those who did not want to participate in the survey were excluded. Ethical clearance was obtained from the Liverpool School of Tropical Medicine Research Ethics Committee (Research Protocol 11.89R).

### Field survey preparation

A sensitization and preparatory meeting was conducted about one week prior to the start of the survey. District and sub-district managers of both health and education sectors, and primary health care staff attended the meeting. Details of the objectives and procedures of the surveys were shared in this meeting, and a suitable time schedule was developed in consultation with both departments. Prior to visiting the school and starting the survey, the team visited the Public Health Centres to brief the medical officer and staff, who also took part in and supported the surveys.

Each year, field teams were selected to conduct the TAS activities, and they visited all the districts and completed all the surveys. Each team consisted of two members from the LF Programme, and two members from the local Public Health Centres. Taking into consideration the distance between the selected schools and logistics, the teams visited two to four schools per day depending on the number of teams sent from Dhaka. The two teams used the same vehicle and the two schools nearest each other were selected to make it convenient and more cost effective. On average a TAS activity took one week to complete and cost approximately US$6000 per EU.

### TAS activity

On the day of survey, the team visited the ‘selected’ school, re-briefed the head master and other teachers of the school about the purpose and objectives of the TAS. A room within the school was then selected and set up to conduct the survey. A teacher worked with the team to organise students to be tested and to provide support to the TAS team.

All the 1^st^ and 2^nd^ grade students were seated in their class rooms and given serial numbers on a laminated piece of paper. The students bearing the numbers that matched the random numbers (generated by SSB), were selected and guided for blood sampling. Each student was gently explained the purpose of the blood sampling in simple language. Those students who were very fearful of blood sampling, even after explanation, were excluded from the survey. If any random number fell on a student of higher age (>7years) of the same grade the respective student was also excluded. However, the proportion of such students was negligible. In his/her place, the child with the next number was selected for sampling. Students were called one by one for finger pricking blood sampling using a disposable needle. Blood was collected into the capillary tube and then put directly on the immunochromatographic test (ICT) card to detect the presence of *W*. *bancrofti* antigen. The ICT card was manufactured by Binax NOW (Alere Inc., Scarborough, ME; http://www.alere.com/en/home/product-details/binaxnow-filariasis.html), and a positive antigen control was used to test the validity before the start of TAS. Each child was asked to rest for 5 minutes after giving the blood sample and then proceed back to the class.

**Fig 4:**
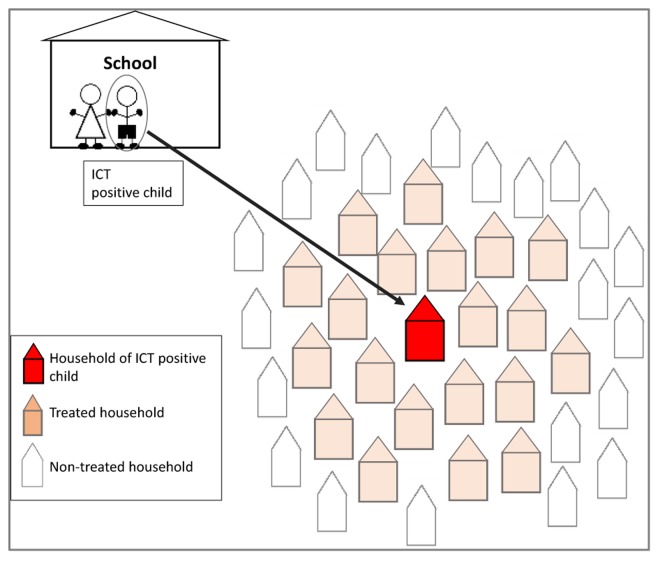
Method for community follow-up and treatment.

For each student, the name, sex and age were recorded on the ICT card and on a data form. The ICT test and reading of the result was done strictly in accordance with the guidelines provided by the manufacturer. The results of the test were noted against each child’s name and directly on the ICT card with a permanent marker pen. ICT positive students, their families and 20 households in close proximity were treated with single dose of albendazole and DEC (diagrammatically shown in Fig 4), and provided with information on the transmission and associated risk factors of LF. As there were no formal guidelines, the decision for the LF Programme to treat 20 households was arbitrary and based on the i) high probability of transmission within the positive household and neighbouring households, and ii) tendency for the main *Culex* sp. mosquitoes to feed and breed in close proximity. The details of the positive student and their family were kept confidential during this localised follow-up MDA activity. The TAS data from each school were entered into an electronic database. All results were reported by the LF Programme to the local Public Health Centres, the surveyed schools and Ministry of Education.

### Data analysis and mapping

For each endemic district a summary of the population size, baseline Mf prevalence, MDA start year, number of MDA rounds, reported and verified coverage rates and information related to the SSB calculations were collated and summarised. For each low endemic district, the population size and baseline Mf prevalence were collated. The TAS results were tabulated and the distribution of ICT positive students in endemic and low endemic districts mapped at sub-district level using geographical information software ArcGIS 10.2 (ESRI, Redlands, CA), to better understand the geographical patterns of potential residual infection or persistent transmission at a finer scale. In Bangladesh, the main sub-district administrative units are upazilas and unions. In total there are 492 upazilas (approximately 7.68 upazilas per district) and 4,501 unions (approximately 70.33 unions per district). As only about 30 schools per EU are sampled under TAS, these schools only represent a proportion of the unions, which will vary between EUs, and also limited sub-district level spatial analysis.

## Results

### TAS for endemic districts (received MDA)

A summary of the TAS results for endemic districts is found in Table 2, and highlights the overall higher baseline prevalence rates and number of MDA rounds in the northern Rangpur Division. The range of reported and independently verified MDA coverage rates is also summarised and indicates overall high coverage across all districts in most years (See S1 File for details). For the first five districts to conduct TAS in 2011, the two districts of Dinajpur and Rajshahi (IUs for MDA implementation) were divided into two EUs each as the population exceeded the recommended 2 million. This resulted in seven EUs for the first TAS and these same divisions were also used for second and third TAS–known as TAS 2 and TAS 3. Similarly, for the TAS activities conducted in 2012, 2013, 2014 and 2016, the large population districts were divided into two EUs each (Sirajganj and Pabna in 2012; Barisal in 2013, and Kurigram and Rangpur in 2014 and 2016), which resulted in seven, six and six EUs being assessed in these years respectively.

Since 2011, a total of 59 TAS activities (TAS 1 in 26 EUs; TAS 1 repeated in 2 EUs; TAS 2 in 24 EUs; TAS 3 in 7 EUs) have been conducted in 26 EUs across the 19 endemic districts that had received MDA. A total of 99,148 students from 1,801 schools have been tested. The number of primary schools, and number of 1^st^ and 2^nd^ grade students in each EU ranged from 328 to 1,109, and from 35,137 to 93,500 respectively. Based on the SSB calculations, the number of schools selected in each EU ranged from 30 to 36, with sample sizes of 1,692 students and a critical cut-off of 20 for every EU, except for Meherpur and Jhalokathi where 1,556 students were tested and had a critical cut-off of 18 each (Table 2). All EUs passed the critical cut-off with the exception of Rangpur-B in 2014, which repeated two more rounds of MDA together with adjacent Rangpur-A (details are described below in the Rangpur District TAS overview section). In total, 112 students were found to be ICT positive across the three TAS activities (Table 3). The majority of ICT positive students were found in the highly endemic northern Rangpur Division (n = 94; 83.9%), compared with the central western Rajshahi Division (n = 13; 11.6%) and the southern Barisal Division (n = 5; 4.5%). A significant positive correlation was found between district baseline Mf measures and the number of ICT positive students found in TAS 1 (Pearson’s correlation coefficient r = 0.464; p = 0.045). There was no significant difference between the number of male (n = 59) and female (n = 53) students found to be ICT positive.

**Table 3:** Number of ICT positive students by division, EU and sex of student.

Divisions (Region)	EU	Sex		Total
**Rangpur (North)**		**F**	**M**	
	Dinajpur A	2	5	7
	Dinajpur B	6	6	12
	Kurigram A	1	1	2
	Lalmonirhat	8	4	12
	Nilphamari	5	9	14
	Rangpur A	7	7	14
	Rangpur B	17	13	30
	Thakurgaon		3	3
**Rajshahi/Khulna (Central)**				
	Chapainawabganj	3	1	4
	Pabna	2	1	3
	Rajshahi A		1	1
	Rajshahi B		4	4
	Shirajganj A		1	1
**Barisal (South)**				
	Barguna	1	1	2
	Jhalokathi	1		1
	Pirojpur		2	2
	**Total No. Cases**	**53**	**59**	**112**

**Fig 5:**
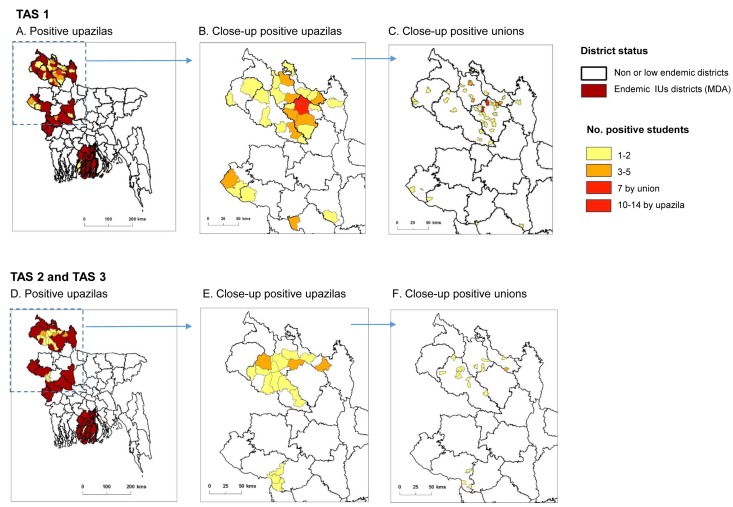
Maps of TAS endemic districts and ICT positive upazilas and unions. TAS 1: A. Positive upazilas. B. Close-up positive upazilas. C. Close-up positive unions. TAS 2 and TAS 3: D. Positive upazilas. E. Close-up positive upazilas. F. Close-up positive unions.

For TAS 1, there were 24 EUs from 18 endemic districts (IUs) that passed and stopped MDA. Of these EUs, a total of 40,336 students from 733 schools were tested, with 41 students found to be ICT positive (Table 2). The majority of ICT positive students were from Nilphamari (n = 9 students; 5 schools), Lalmonirhat (n = 8 students; 6 schools), Dinajpur-B (n = 6 students; 5 schools), Chapainawabganj (n = 4 students; 4 schools), Pabna-B (n = 3 students, 1 school), and Thakurgaon (n = 3 students, 3 schools). There was no difference between males (n = 20) and females (n = 21), and most were aged 6 years (n = 19) or 7 years (n = 20) with two aged 8 years. TAS 1 endemic districts requiring MDA with the related upazilas that reported ICT positive students are shown in Fig 5A. Close-up maps of the ICT positive upazilas and unions in northern region are shown in Fig 5B and Fig 5C respectively, and highlight that the majority of upazilas and unions show few or no positive students, except Rangpur and Gangachara upazilas. The union map specifically highlights the geographically sparse distribution and reduction in endemicity with only two unions reporting more than two positive cases in Rangpur.

For TAS 2, there were 24 EUs from 18 districts that passed with results below the critical cut-off. A total of 40,336 students from 733 schools were tested, with 25 students found to be ICT positive. The majority of ICT positive students were from Dinajpur-A (n = 5 students; 4 schools), Dinajpur-B (n = 5 students, 3 schools), and Nilphamari (n = 5 students; 4 schools). Of the 25 positive students, 18 were males and 7 females, and were mainly aged 6 years (n = 14), or 7 years (n = 8) with two aged 5 years and one aged 8 years. For TAS 3, there were seven EUs from five districts that passed with results below the critical cut-off. A total of 11,708 students from 215 schools were tested, with only two students found to be ICT positive, who were from Dinajpur-A (n = 1; 7 year old male student, 1 school) and Dinajpur-B (n = 1; 6 year old female student, 1 school). A map of the TAS 2 and TAS 3 districts and positive upazilas are shown in Fig 5D. Close-up maps of the ICT positive upazilas and unions in northern region are shown in Fig 5E and Fig 5F respectively, and further highlight the geographically sparse distribution and reduction in endemicity with no union reporting more than two positive students across the region.

### Rangpur district TAS overview

**Fig 6:**
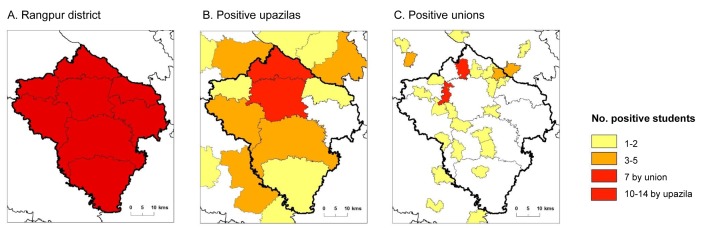
Rangpur district and regional ICT positive upazilas and unions for TAS 1. A. Rangpur district. B. Positive upazilas. C. Positive unions.

Rangpur district had a 10% Mf baseline prevalence rate, and since 2005 had received nine rounds of MDA with coverage rates ranging from 66.3% to 95.9% and pre-TAS measures from 0.05–0.1% (Table 2). The EU Rangpur-B failed TAS 1 with 27 of 1,692 students tested from 30 schools found to be ICT positive (males = 12; females = 15). Most cases were found in Gangachara (n = 14) and Rangpur (n = 10) upazilas. The adjoining EU Rangpur-A, which found 11 ICT positive students (males = 4; female = 7), primarily in Badarganj (n = 4) and Mithapukur (n = 5) upazilas, and although passed TAS, was recommended to conduct two further rounds of MDA together with Rangpur-B. The Rangpur EUs and the upazila boundaries are shown in Fig 6A. The number of ICT positive students by upazila for this failed TAS 1 is shown in Fig 6B, highlighting the proximity of Gangachara and Rangpur upazilas. The distribution of ICT positive students by union are shown in Fig 6C, highlighting two focal locations where seven ICT positive students each were reported in Barabil and Mominpur unions, which are adjacent to the large urban areas of Rangpur upazila. Since the failed TAS 1, MDA campaigns with enhanced social mobilisation activities have been conducted in 2014 and 2015. The TAS 1 was repeated in Rangpur-A and Rangpur-B in November 2016 (1,692 students tested from 30 schools per EU), with three ICT positive students found in each EU. In Rangpur-A, ICT positive students were from Mithapukur (n = 2); and Badargani (n = 1) upazilas, and in Rangpur-B from Rangpur (n = 3) and Kaligonj (n = 1) upazilas. All positive students were from different schools.

### TAS- low endemic districts (not received MDA)

Of the 15 low endemic districts, seven districts—Bogra, Narayanganj, Narshingdi, Gazipur, Jamalpur and Habiganj, and peri-urban Dhaka–had large populations and were divided into two EUs each. This resulted in a total of 22 EUs that were assessed in 2014 and 2015 (Table 4). The number of primary schools, and number of 1^st^ and 2^nd^ grade students for each of the EUs ranged from 387 to 4,071, and from 21,069 to 108,393 respectively. Based on the SSB calculations, the number of selected schools visited ranged from 30 to 33. The sample size was 1,692 (critical cut-off = 20) for all EUs, except for Bandarban where it was 1,552 (critical cut-off = 18), Narail where it was 1,556 (critical cut-off = 18), and Gazipur (A and B) where it was 1,684 (critical cut-off = 20) (Table 4).

**Table 4:** Summary measures and TAS results for the 15 low endemic districts (not received MDA).

Districts	Pop. size in million 2011 census	Baseline year ICT	ICT results % (no. test)	Follow-up Year MF (no. sites)	MF* % (no. test)	Sample size	N0. of schools tested	No of ICT +ves
Bagerhat	1.46	2002–04	13% (32)	2006–10	0%	1692	33	1
				4 sites	(2000)			
Narail	0.72	2002–04	2.4% (125)	2006–10	0%	1556	30	1
				4 sites	(2000)			
Feni	1.42	2002–04	0% (125)	2005–09	0	1692	30	0
				4 sites	(2000)			
Laxmipur	1.71	2002–04	0% (125)	2006–09	0	1692	30	1
				2 sites	(2000)			
Bandarban	0.38	2002–04	0% (125)	2007–10	0	1552	30	0
				4 sites	(2000)			
Bogra- A	3.37	2002–04	0% (50)	2009	0	1692	30	1
Bogra–B				2 sites	(1000)	1692	30	0
Munshiganj	1.42	2002–04	8% (98)	2007–09	0%	1692	30	0
				4 sites	(2000)			
Jhenaidaha	1.76	2002–04	0% (125)	2006–10	0%	1692	30	0
				4 sites	(2000)			
Gopalgani	1.15	2002–04	15% (100)	2009	0%)	1692	30	0
				2 sites	(2000)			
Narayanganj–A	2.90	2002–04	0% (100)	2007–09	0%	1692	30	0
Narayanganj—B				4 sites	(2000)	1556	30	0
Narshingdi–A	2.20	2002–04	2.5% (80)	2006–09	0%	1692	30	0
Narshingdi–B				4 sites	(2000)	1692	30	0
Gazipur–A	3.33	2002–04	5% (52)	2007–09	0%	1684	30	0
Gazipur–B				4 sites	(2000)	1684	30	0
Jamalpur–A	2.27	2002–04	4% (30)	2006–09	0%	1692	30	0
Jamalpur–B				4 sites	(2000)	1692	30	0
Habiganj–A	2.06	2002–04	6% (100)	2007–10	0%	1692	30	0
Habiganj–B				4 sites	(2000)	1692	30	0
Dhaka (peri-urban)—A	1.79	2002–04	14% (147)	2007**	0.05	1692	30	0
Dhaka (peri-urban)—A				24 sites in city	(10,360)	1692	30	0
**TOTAL**	**27.94**					**36932**	**663**	**4**

* 500 individuals sampled for Mf at each sentinel site.

** Dhaka city– 24 sites tested across the city with 5 positives individuals found in 5 different locations, including Mir Hazi, Walbhanga, Korail, Mirpur and Tejgaon informal settlements in peri-urban areas.

All EUs from the low endemic districts passed the TAS critical cut-off. In total 36,932 students from 663 schools were tested across the 22 EUs, with only four students found to be ICT positive. The students testing ICT positive were from four different EUs including Bagerhat, Narail, Laxmipur and Bogra-A. Of the four ICT positive students, there was one male and three females, with one aged 6 years, one aged 10 years, and two aged 7 years.

## Discussion

This paper presents results from the largest scale up of post-MDA surveillance activities reported in any of the 73 LF endemic countries in the world [22]. The TAS results and the maps presented in this paper have shown a significant reduction in prevalence and geographical distribution of infection since the inception of MDA, with approximately 70 million people across Bangladesh now considered to be at very low or no risk of LF infection. This is a major step towards the national elimination goal of 2020, and key contribution to the efforts of GPELF and its specific goal to interrupt transmission [1,23]. Further, the paper presents a detailed account of the WHO TAS implementation [15], and highlights its potential use in low endemic areas, which may be applicable for other countries in certain epidemiological situations. Addressing low endemic areas or re-evaluating endemicity using a decision making prevalence survey based on probability sampling [24], is becoming increasingly important as countries need to provide nationwide evidence that LF is no longer a public health problem as part of the elimination validation and dossier requirements.

The overall success of the LF Programme in implementing MDA, reducing prevalence and scaling up TAS activities as an endgame surveillance strategy may be attributed to many key determinants of success [25,26]. Factors highlighted in the review by Kyelem et al. [25] are grouped as biological/ epidemiological/therapeutic, economic/political/social and programmatic operational effectiveness, and the most prominent factors identified that relate to Bangladesh include i) the initial level of LF endemicity ii) MDA drug regime and iii) population compliance. Bangladesh had generally low transmission levels at baseline (i.e. pre-MDA) with the majority of Mf and ICT prevalence rates <15%, further it used the drug combination of albendazole and DEC, which is considered highly effective against the *W*. *bancrofti* parasite [27,28], and the programme was able to achieve high MDA coverage rates >70% across the majority of endemic districts.

Other positive influencing factors for Bangladesh include good administrative development and health system infrastructure, relative political stability, strong political commitment and financial support, strong programme management leadership, heightened awareness of morbidity in the endemic areas, which helped to increase drug compliance and importance of disease elimination [13]. The LF Programme has also been operationally effective in many standard practices including advocacy, training drug distributors, involving community leaders in social mobilization, coordinating drug logistics with predictable MDA schedules. The LF Programme is integrated with the National Soil Transmitted Helminth (STH) Programme that implements MDA twice yearly to all primary school-children, which has also helped to increase the community knowledge and awareness of helminths across the country.

Furthermore, the Bangladesh LF programme managers have collaborated well with partnering organisations, who have been able to provide key financial and technical support. This has helped to address challenges and maintain momentum towards the elimination goal. For example, the extensive scale up of TAS activities have been achieved by two dedicated TAS teams who have worked year round with the financial support from international donors. In addition, specific technical support has been provided to develop detailed maps of prevalence data and all TAS results to highlight potential ‘hotspots’ of persistent transmission that need targeting with further investigations, interventions and surveillance. The LF Programme has also engaged in operational research to enhance the standard monitoring and evaluation activities with key findings and success stories presented internationally, including surveillance strategies and the use of TAS for low endemic areas [16,29,30].

Notwithstanding the positive factors outlined above, the LF Programme has had several challenges, particularly in the northern region where several districts received many more MDA rounds than recommended. The reason for the high number of MDA rounds was related to two main factors. First, the lack of a formal strategy with defined thresholds of ‘when to stop MDA’, meant the programme kept implementing until the TAS guidelines were released in 2010 [15]. Second, sentinel site data showed evidence of persistent infection in a number of districts up until 2013, and the recent failure of TAS 1 in Rangpur district in 2014. The reason for the persistence is not clear, but may be related to localised demographic or environmental characteristics such as poverty [31], peri-urbanisation [32], presence of *Culex* spp. mosquito breeding sites [6], slightly lower coverage rates, which are currently being investigated in more detail to better prepare for future surveillance. Rangpur district failing TAS was a setback for the LF programme with the elimination target being realigned, and a further two rounds of MDA implemented in November 2014 and 2015. To increase coverage during these MDA rounds, mapping of coverage rates and TAS results at sub-district level helped to identify and target problem areas, which helped the repeated TAS 1 to succeed and pass the critical cut-off in November 2016.

The TAS maps presented in this paper highlight that the persistent areas are quite focal with only a small number of upazilas and unions reporting a higher number of ICT positive students, with no specific gender or primary school identified to be at increased risk. While the distribution of positive students was found to be relatively geographically sparse across each district, an overall positive relationship between baseline Mf measures and the number of ICT positive students during the first TAS was found. This is despite the different diagnostics used in different surveyed populations at different spatial resolutions. A broad regional association was also evident with the highest number of TAS ICT positive students found in the northern Rangpur Division, which reported the highest historical and baseline Mf rates (i.e. pre-MDA)[7,12].

These results support the notion that EUs in higher risk regions, may be more likely to fail TAS and/or have areas of persistent residual infection i.e. ‘hotspots’. These and other key associations such as MDA frequency, coverage and adherence patterns as well as vector control, are being examined with modelling methods in Asia and Africa [33–37] and these may help predict hotspots. They may also help to determine appropriate diagnostic tools and surveillance strategies, with assessments of the TAS EU size also being considered as countries move closer to the elimination goal [38–41]. This is important as all districts in Bangladesh need to pass three TAS activities over a 5–6 year period, and will now be using the new rapid diagnostic Alere Filariasis Test Strip (FTS) Alere, Scarborough, ME, United States, which is more sensitive to detect Ag and as a consequence may detect more positive students [42].

The most significant activity and potential challenge for the LF programme now is to develop and maintain a sustainable long-term surveillance system that is integrated into existing health infrastructure and other ongoing programmatic activities [43]. It will be crucial that the system is tailored to detect the risk of recrudescence, which may be determined from a combination of demographic information, programmatic data such as baseline prevalence, sentinel sites, TAS results as well as the local health infrastructure capacity. For Bangladesh the northern Rangpur Division is clearly a priority surveillance area with vigilance also required for migrant populations, in international border areas and adjacent non- or low endemic districts as they are vulnerable if there is high movement of untreated people [29,30]. Ramaiah et al. [44] highlights key categories of migration which may have implications for LF elimination efforts, including i) migration from endemic areas to non-endemic areas, ii) migration from endemic rural areas to endemic urban areas, iii) migration from endemic areas to the areas that achieved control/elimination of LF, and iv) trans-border migration.

The LF Programme aims to integrate seroprevalence surveillance with its current morbidity management and disability prevent activities, which are scaling up to address the needs of approximately 40,000 people with clinical manifestations, most of whom are in the northern areas. The programme also plans to link with the STH programme as it expands to include biannual treatments to all secondary school children. A passive detection method in hospital laboratories across the country is also being assessed. Work is underway to refine this comprehensive surveillance system so that the programme is able to readily respond to any potential hotspots detected in the future, and also to provide evidence that Bangladesh has successfully addressed the essential national, South-East regional and GPELF requirements and eliminated LF as a public health problem [2–4,21,29].

## Supporting information

S1 Checklist STROBE checklist. (DOC)

S1 File District reported and independently verified MDA coverage rates 2009–2014. (DOCX)
