# Supplementary material for: The significant scale up and success of Transmission Assessment Surveys 'TAS' for endgame surveillance of lymphatic filariasis in Bangladesh: One step closer to the elimination goal of 2020
Source: PLoS Negl Trop Dis. 2017 Jan 31;11(1):e0005340. doi: 10.1371/journal.pntd.0005340 (PMC5302837; doi:10.1371/journal.pntd.0005340)
Supplement: S1 File — (DOCX) [file pntd.0005340.s002.docx]

**District reported and independently verified MDA coverage rates 2009 - 2014**

| **Division** | **District** | **2009 reported** | **2009 verified** | **2010 reported** | **2011 reported** | **2011 survey** | **2012 reported** | **2012 verified** | **2013 reported** | **2013 verified** | **2014 reported** | **2014**  **Verified** |
| --- | --- | --- | --- | --- | --- | --- | --- | --- | --- | --- | --- | --- |
| Rangpur | Panchagar | 95.8 | 92.4 | 94.8 | 75.1 | 96.2 | 93.0 | 96.7 |  |  |  |  |
|  | Nilphamari | 99.7 |  | 93.3 | 80.5 | 86.3 | 86.9 | 93.7 | 83.0 | 89.5 |  |  |
|  | Lalmonirhat | 92.2 | 88.8 | 93.5 | 82.6 | 93.7 | 89.3 | 94.1 | 89.3 | 91.3 |  |  |
|  | Kurigram | 91.9 | 86.8 | 92.3 | 84.5 |  | 88.7 | 78.8 | 86.6 | 86.6 |  |  |
|  | Thakurgaon | 93.6 | 87.4 | 91.5 | 86.9 | 90.3 | 86.9 | 74.9 |  |  |  |  |
|  | Dinajpur | 96.9 |  | 89.7 |  |  |  |  |  |  |  |  |
|  | Rangpur | 95.9 |  | 87.8 | 83.4 | 82.7 | 77.4 | 66.3 | 75.6 | 74.0 | 81.7 | 72.6 |
| Rajshahi | Chapainawabganj | 98.1 | 82.6 | 92.0 | 88.9 |  | 93.8 |  |  |  |  |  |
|  | Rajshahi | 82.0 |  | 91.9 |  |  |  |  |  |  |  |  |
|  | Sirajganj | 92.7 | 80.4 | 92.6 | 79.4 |  |  |  |  |  |  |  |
|  | Pabna | 99.0 |  | 92.0 | 88.4 |  |  |  |  |  |  |  |
| Khulna | Meherpur | 93.8 | 87.6 | 90.9 |  |  |  |  |  |  |  |  |
|  | Chuadanga | 92.4 | 84.5 | 96.0 | 78.1 |  |  |  |  |  |  |  |
|  | Kushtia | 86.0 |  | 95.3 | 96.3 |  |  |  |  |  |  |  |
| Barisal | Barguna | 94.5 |  | 95.4 |  |  |  |  |  |  |  |  |
|  | Patuakhali | 92.8 | 87.1 | 96.8 |  |  |  |  |  |  |  |  |
|  | Pirojpur | 93.4 |  | 92.4 | 57.8 |  |  |  |  |  |  |  |
|  | Jhalikhati | 98.3 |  | 91.3 | 74.4 |  | 76.8 |  |  |  |  |  |
|  | Barisal | 88.9 | 80.1 | 92.9 | 79.9 |  | 88.1 |  |  |  |  |  |
|  |  |  |  |  |  |  |  |  |  |  |  |  |
